# The Burt Case

**Published:** 1898-06

**Authors:** 


THE BURT CASE.—Readers of the JOURNAL will remember that W. E. Burt, youngest son of the late Dr. W. J. Burt, of Austin, long while Secretary of the Texas State Medical Association, was convicted of the murder of his wife and children, and that insanity was set up as a defense. Every resource known to the able lawyers entrusted with his defense was exhausted to save his neck, but all in vain. Young Burt was executed in the county jail at Austin, May 27, ult. He died a stoic,—mounted the gallows bravely; stepped on the trap and gave the signal to

---

*No, it wasn't; it was Gwinn, one C. L. Gwinn, of Galveston, the party who insulted and drove from his office a young lady who had made the mistake of soliciting his subscription to a journal of which her father was editor, because he had the execrable taste to not like said editor.—ED.

spring it, without the tremor of an eyelash. His last statement is given below; it is a curious study:

BURT'S DYING STATEMENT.

AUSTIN, TEXAS, May 27, 1898.

*To Whom it May Interest:*

Realizing that before noon of this day I will be hung by the neck until I am dead, and that ere this is given to the public I will be dead, I desire to say a few words. A deliberate lie in face of such a death is unpardonable either on earth, in hell or heaven. Where I go I soon shall know.

But now and forever I solemnly swear that my own hand did not kill my darling wife and sweet little Lucile and Elenor. I swear that I did not know that they were to be murdered. I swear that I was not an accessory or in any way connected with their murder. But I also solemnly swear that I knew before I left Austin who was guilty. And further, that in my rage no other idea save that of retribution ever possessed me.

Again, my firm belief is that ere another dies, who is now unknown to me, he will acknowledge that he was the schemer and originated this plan of revenge on me, knowing that I would be accused, rather than taking my own life as satisfaction to him.

I solemnly swear that he who slew my loved ones is now dead and has been for many, many months, and that I was his slayer, and with my own eyes saw vengeance so full and complete, and that every bone and ligament of his carcass was absolutely destroyed.

Again, that no thought of allowing lawful justice was ever for a moment considered by me.

And in my desire for vengeance, the human fiend he made of me has never for one second regretted my action. I am glad of it.

And in my haste and rage I destroyed everything that would have been of assistance to me in establishing my innocence. I have not a single regret. I never waver for a moment when my mind is made up.

I am unrelenting in the pursuance of any object and never let up until I finish. When I do a thing I consider every phase of the case, and if called to account for my actions I am prepared to accept the situation with as little complaining as possible, face the new foe with my banner of "satisfaction" unfurled to the breeze until it and I sink into everlasting oblivion.

This is all I have to say. One by one as you journey past the valley of death you will find that in this sworn death statement, I have not lied. Respectfully,

W. E. BURT.

Subscribed and sworn to before me on this 27th day of May, A. D. 1898. [Seal] JNO. W. HORNSBY,
Clerk County Court, Travis County, Texas.

Additional:—Rumors have come to my hearing questioning the feasibility of some one else having written or composed the article that I was pleased to have published on last Sunday.

I will say that no living human being ever knew one word or idea advanced which was contained in that article but myself. I requested the paper of Mr. Hughes on Friday morning, and wrote said article Friday evening and night. Also sent word to the reporter of my desire to see him and I delivered the manuscript to him [Asher Smoot] on Sunday evening at 3:30.

*I never require assistance. If I cannot accomplish anything it must go undone.* W. E. BURT.

—*From the Austin Daily Statesman.*

[Burt was about 29 years of age.—ED.]

